# A TNF receptor 2 agonist ameliorates neuropathology and improves cognition in an Alzheimer’s disease mouse model

**DOI:** 10.1073/pnas.2201137119

**Published:** 2022-08-29

**Authors:** Natalia Ortí-Casañ, Inge S. Zuhorn, Petrus J. W. Naudé, Peter P. De Deyn, Pauline E. M. van Schaik, Harald Wajant, Ulrich L. M. Eisel

**Affiliations:** ^a^Department of Molecular Neurobiology, Groningen Institute for Evolutionary Life Sciences, University of Groningen, Groningen 9747 AG, Netherlands;; ^b^Department of Biomedical Engineering, University of Groningen, University Medical Center Groningen, Groningen 9713 AV, Netherlands;; ^c^Department of Neurology and Alzheimer Center, University of Groningen, University Medical Center Groningen, Groningen 9713 AV, Netherlands;; ^d^Department of Biomedical Sciences of Cells & Systems, Section Molecular Neurobiology, University of Groningen, University Medical Center Groningen, Groningen 9713 AV, Netherlands;; ^e^Department of Internal Medicine II, University of Würzburg, Würzburg 97070, Germany

**Keywords:** Alzheimer’s disease, TNF, neuroinflammation, TNFR2 agonist, J20 mouse model

## Abstract

Tumor necrosis factor-α (TNF-α) is a pleiotropic, proinflammatory cytokine related to different neurodegenerative diseases, including Alzheimer’s disease (AD). Although the linkage between increased TNF-α levels and AD is widely recognized, TNF-α–neutralizing therapies have failed to treat AD. Previous research has associated this with the antithetic functions of the two TNF receptors, TNF receptor 1, associated with inflammation and apoptosis, and TNF receptor 2 (TNFR2), associated with neuroprotection. In our study, we investigated the effects of specifically stimulating TNFR2 with a TNFR2 agonist (NewStar2) in a transgenic Aβ-overexpressing mouse model of AD by administering NewStar2 in two different ways: centrally, via implantation of osmotic pumps, or systemically by intraperitoneal injections. We found that both centrally and systemically administered NewStar2 resulted in a drastic reduction in amyloid β deposition and β-secretase 1 expression levels. Moreover, activation of TNFR2 increased microglial and astrocytic activation and promoted the uptake and degradation of Aβ. Finally, cognitive functions were also improved after NewStar2 treatment. Our results demonstrate that activation of TNFR2 mitigates Aβ-induced cognitive deficits and neuropathology in an AD mouse model and indicates that TNFR2 stimulation might be a potential treatment for AD.

## Significance

Neuroinflammation, a major hallmark of Alzheimer’s disease (AD), has recently emerged as a potential therapeutic target. The important role of TNF-α, a master pro-inflammatory cytokine, in AD is well known. However, anti–TNF-α therapies have failed to treat AD, possibly due to the opposing functions of its receptors TNFR1 (neurodegeneration) and TNFR2 (neuroprotection). Thus, in this study, we investigated the effects of activating TNFR2 with a TNFR2 agonist in an AD mouse model. We show that stimulation of TNFR2 mitigates neuropathological features in AD mice by drastically decreasing the production rate of amyloid β and increasing its clearance by glial cells, resulting in an improvement of memory functions. Therefore, TNFR2 activation may be valuable as potential therapy for AD.

Tumor necrosis factor-α (TNF-α) is a master proinflammatory cytokine involved in the regulation of innate and adaptive immunity (1). TNF-α plays a crucial role in various autoimmune and neurological disorders, including Alzheimer’s disease (AD) (2, 3). TNF-α–neutralizing therapeutics have been approved for the treatment of different inflammatory and autoimmune diseases, like rheumatoid arthritis or plaque psoriasis (4). However, the treatment of neurological disorders with TNF-α–neutralizing drugs led to inconclusive or even detrimental results (5–8). The latter could be explained, at least partly, by the different functions of the two receptors of TNF: on the one hand, stimulation of cytotoxic and strongly inflammatory pathways by TNF receptor 1 (TNFR1) in response to soluble TNF (sTNF) or membrane TNF (mTNF) or, on the other hand, activation of TNF receptor 2 (TNFR2) by mTNF eliciting pro- and antiinflammatory effects but also neuroprotective functions and tissue regeneration (9, 10).

As a consequence, several studies aimed at selective targeting of TNFR1 or TNFR2, instead of completely inhibiting TNF-α. First, targeting TNFR1 by using specific TNFR1 antagonists or sTNF inhibitors resulted in amelioration of inflammation and apoptosis in various in vivo neurodegenerative disease models, such as models of multiple sclerosis (11, 12), Parkinson’s disease (13), and AD (14, 15). Second, targeting TNFR2 by specific TNFR2 agonists exerted an enhancement in neuroregeneration and tissue homeostasis in vitro (16) as well as in in vivo models (17). Therefore, specific targeting of TNFR1 and/or TNFR2 offers a promising new therapeutic avenue.

However, clinical and preclinical studies on the effect of specific therapeutic targeting of TNFR2 in AD are lacking. It is acknowledged that during AD, the deposition of Aβ plaques, one of the main hallmarks of AD pathogenesis, occurs as a consequence of an imbalance between Aβ production and clearance (18). The β-secretase 1 (BACE-1) is mainly responsible for the production of toxic Aβ peptides, whereas uptake and degradation of these peptides are achieved by glial cells in the central nervous system (CNS), such as microglia and astrocytes (19, 20). Microglia are the resident macrophages of the CNS and their function is to constantly survey their environment and react to any detected insult, such as tissue damage or pathogen infection (21). Microglia are also involved in tissue repair and maintaining brain homeostasis. Moreover, microglia have been found to be essential for Aβ clearance in AD mouse models (20). It has been reported that in the context of AD, BACE-1 expression is increased, and the presence of Aβ plaques impairs the phagocytic activity of microglia (22, 23). These events could lead to an overproduction of Aβ accompanied by reduced Aβ clearance, which may partly lead to the observed pathogenesis of AD.

Previous studies have proven that TNFR2 activation is neuroprotective in different disease models; however, it remains to be established whether TNFR2 stimulation has a protective effect against Aβ-mediated neuropathology and amyloid deposition and if this could improve cognitive functions. Therefore, in this study, we tested the hypothesis that selective stimulation of TNFR2 is able to abrogate Aβ-associated neuropathology and cognitive impairments.

## Results

### NewStar2 Improves Cognitive Functions in the J20 Mouse Model.

**Fig. 1.**
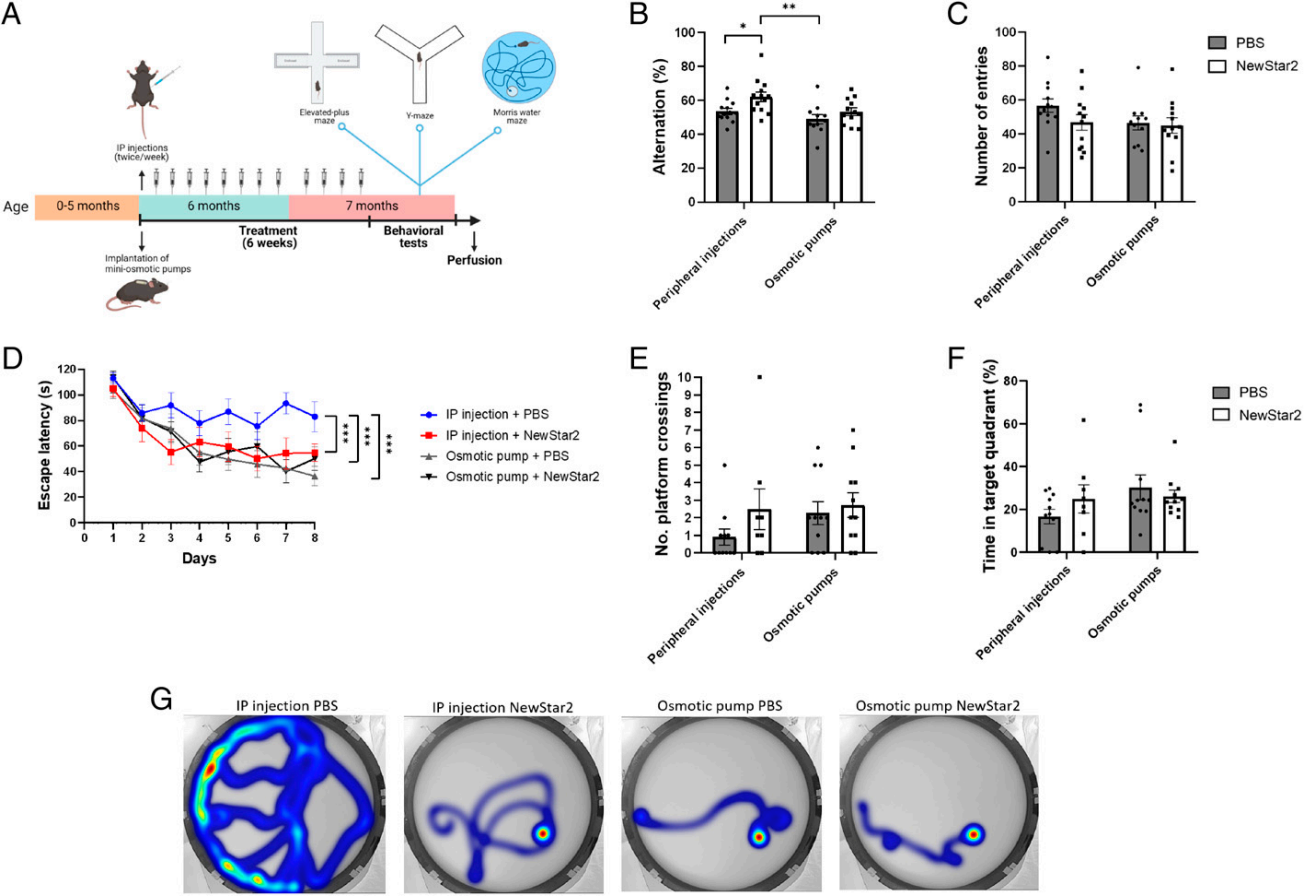
NewStar2 improves working and spatial memory. (*A*) Timeline of experimental procedures. (*B*) Alternation (%) tested in the Y-maze spontaneous alternation (IP injections: PBS, *n* = 13, NewStar2, *n* = 13; osmotic pumps: PBS, *n* = 11, NewStar2, *n* = 12; two-way ANOVA, Bonferroni post hoc analysis: F_1,45_ = 7.81, degrees of freedom [DF] = 1). (*C*) Number of entries in the Y-maze spontaneous alternation (IP injections: PBS, *n* = 13, NewStar2, *n* = 13; osmotic pumps: PBS, *n* = 11, NewStar2, *n* = 12; two-way ANOVA, Bonferroni post hoc analysis: F_1,45_ = 1.88, DF = 1). (*D)* Escape latency(s) during the training phase of the MWM (IP injections: PBS, *n* = 11, NewStar2, *n* = 8; osmotic pumps: PBS, *n* = 11, NewStar2, *n* = 11; two-way repeated measures ANOVA, Bonferroni post hoc analysis: F_3,37_ = 4.37, DF = 3). (*E*) Number of platform crossings during the probe trial (IP injections: PBS, *n* = 11, NewStar2, *n* = 8; osmotic pumps: PBS, *n* = 11, NewStar2, *n* = 11; two-way ANOVA, Bonferroni post hoc analysis: F_1,37_ = 1.19, DF = 1). (*F*) Time in target quadrant during the probe trial (IP injections: PBS, *n* = 11, NewStar2, *n* = 8; osmotic pumps: PBS, *n* = 11, NewStar2, *n* = 11; two-way ANOVA, Bonferroni post hoc analysis: F_1,37_ = 2.41, DF = 1). (*G*) Representative heat maps showing swimming trajectories at day 8 of training. The platform was located in the southeast quadrant. Data are presented as mean ± SEM. *P < 0.05; **P < 0.01; ***P < 0.001. Panel *A* was created with BioRender.com.

To assess the potential effects of the TNFR2 agonist NewStar2 in J20 mice on cognition, NewStar2 was administered either centrally via osmotic pumps or systemically via intraperitoneal (IP) injections. After 6 wk of treatment, we had the mice perform the elevated-plus maze (EPM), spontaneous alternation Y-maze, and Morris water maze (MWM) tests (Fig. 1*A*). In the Y maze, animals treated IP with NewStar2 performed significantly more alternations than did phosphate-buffered saline (PBS)–treated control animals. In the osmotic-pump groups, administration of NewStar2 showed no effect (Fig. 1*B*). No significant differences were found in the number of entries irrespective of the method of NewStar2 administration (Fig. 1*C*). Moreover, administration of NewStar2 both via osmotic pumps and peripheral injections resulted in no significant differences in the percentage of time spent in the center, open, or closed arms in the EPM test (*SI Appendix*, Fig. S1 *B* and *C*).

In the MWM training phase, mice treated IP with NewStar2 had a significantly lower escape latency compared with the animals in the IP PBS control group. No significant difference in the escape latency was found when comparing both osmotic-pump groups. However, the escape latency of the mice of both osmotic-pump groups was equivalent to that of the animals IP treated with NewStar2 (Fig. 1*D*), which was also observed in their swimming trajectories (Fig. 1*G*). In the probe trial performed 24 h after the training phase, there were no statistically significant differences in the number of crossings over the platform position (Fig. 1*E*) or the time spent in the platform quadrant (i.e., the target quadrant) (Fig. 1*F*) across the groups. Nevertheless, mice from both osmotic-pump groups and mice IP treated with NewStar2 had a tendency to perform more platform crossings and spend more time in the target quadrant than did the IP PBS-treated animals.

Moreover, changes in body weight were evaluated. Animals carrying osmotic pumps had an overall increase in body weight growth compared with animals that received peripheral injections (*SI Appendix*, Fig. S1*A*).

Additionally, since NewStar2 was administered to the animals via an osmotic pump for a period of 6 wk, we investigated whether NewStar2 would remain stable during this period of time at the animal’s body temperature (∼37 °C). To this end, a cytotoxicity assay using kym-1 cells was performed prior to the start of the animal experiments. Pretreatment of kym-1 cells with 500 ng/mL NewStar2 for 24 h resulted in a significant decrease in cell viability for both freshly conserved NewStar2 and 6-wk preincubated NewStar2 (*SI Appendix*, Fig. S2). These results confirm that NewStar2 remains bioactive at 37 °C for at least 6 wk.

### NewStar2 Reduces Plaque Load and BACE-1 Expression.

**Fig. 2.**
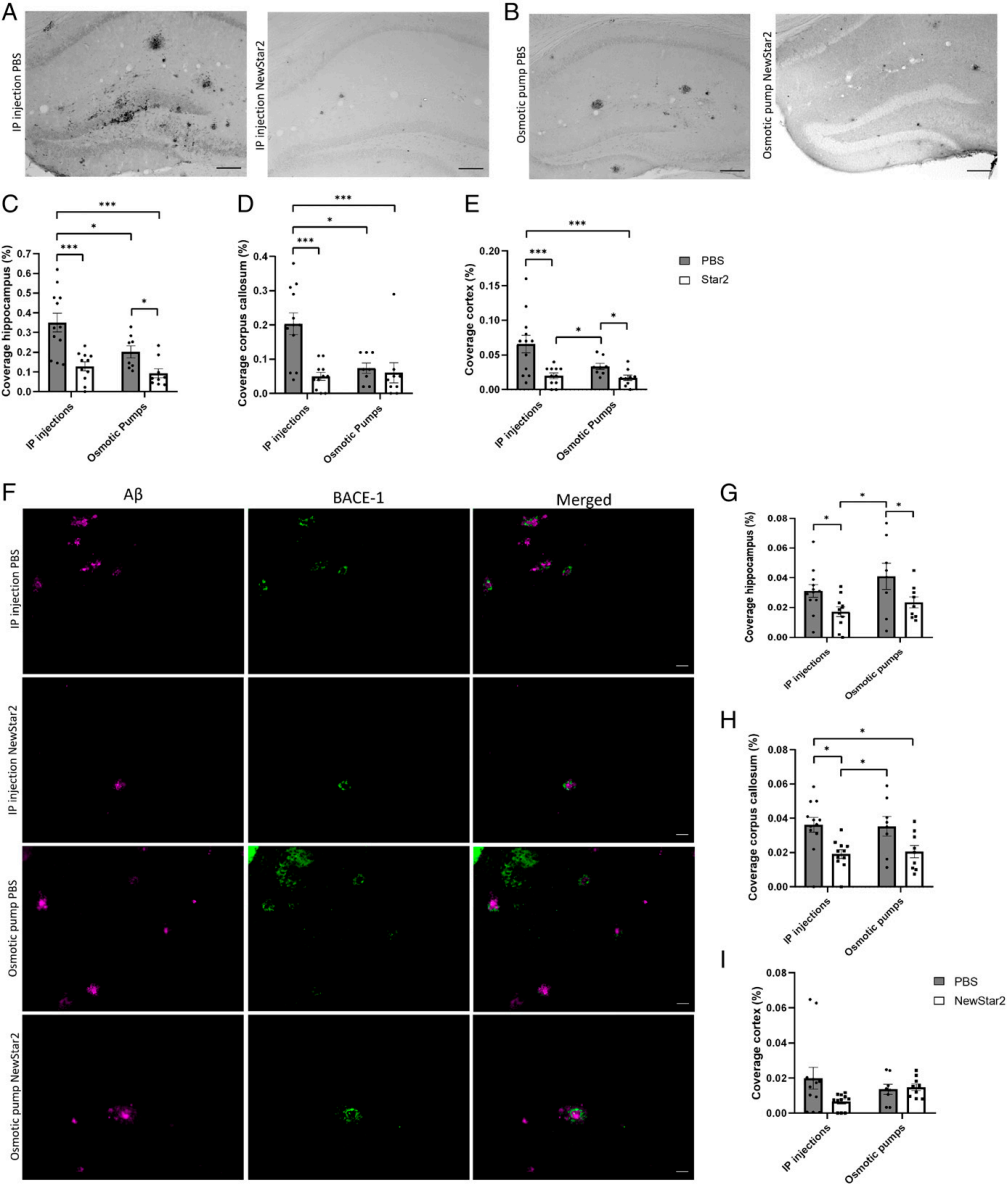
NewStar2 administration decreases Aβ plaque load and BACE-1 expression levels. (*A*) Plaque load after PBS or NewStar2 administration via IP injections. (*B*) Plaque load after PBS or NewStar2 administration via osmotic pumps. Representative images of hippocampus are shown. Scale bar, 100 µm. (*C*–*E*) Quantification of Aβ plaque coverage in hippocampus (IP injections: PBS, *n* = 12, NewStar2, *n* = 11; osmotic pumps: PBS, *n* = 8, NewStar2, *n* = 9; two-way ANOVA, Bonferroni post hoc analysis: F_1,36_ = 6.68, degrees of freedom [DF] = 1) (*C*); corpus callosum (IP injections: PBS, *n* = 12, NewStar2, *n* = 11; osmotic pumps: PBS, *n* = 8, NewStar2, *n* = 9; two-way ANOVA, Bonferroni post hoc analysis: F_1,36_ = 5.58, DF = 1) (*D*); and cortex (IP injections: PBS, *n* = 12, NewStar2, *n* = 11; osmotic pumps (PBS: *n* = 8, NewStar2, *n* = 9; two-way ANOVA, Bonferroni post hoc analysis: F_1,36_ = 4.23, DF = 1) (*E*). (*F*) Sections from tissues of mice treated with PBS compared with those of mice treated with NewStar2 administered via IP injections or osmotic pumps. Sections were stained with 6e10 (magenta) for Aβ plaques and anti–BACE-1 (green) for BACE-1. Representative images of hippocampus are shown. Scale bar, 50 µm. (*G*–*I*) Quantification of BACE-1 coverage in hippocampus (IP injections: PBS, *n* = 12, NewStar2, *n* = 11; osmotic pumps: PBS, *n* = 8, NewStar2, *n* = 9; two-way ANOVA, Bonferroni post hoc analysis: F_1,36_ = 9.64, DF = 1) (*G*); corpus callosum (IP injections: PBS, *n* = 12, NewStar2, *n* = 11; osmotic pumps: PBS, *n* = 8; NewStar2, *n* = 9; two-way ANOVA, Bonferroni post hoc analysis: F_1,36_ = 14.93, DF = 1) (*H*); and cortex (IP injections: PBS, *n* = 12, NewStar2, *n* = 11; osmotic pumps (PBS, *n* = 8, NewStar2, *n* = 9; two-way ANOVA, Bonferroni post hoc analysis: F_1,36_ = 2.09, DF = 1) (*I*). Data are presented as mean ± SEM. *P < 0.05; ***P < 0.001.

To evaluate the effects of NewStar2 on the neuropathology of J20 mice, we investigated the Aβ plaque load after NewStar2 administration. NewStar2 IP administration resulted in a drastic reduction of Aβ plaque load (Fig. 2*A*) measured in hippocampus (Fig. 2*C*), corpus callosum (Fig. 2*D*), and cortex (Fig. 2*E*). Similarly, central application of NewStar2 by osmotic pumps resulted in a reduction in Aβ plaque load (Fig. 2*B*) observed in hippocampus (Fig. 2*C*) and cortex (Fig. 2*E*), but not in the corpus callosum (Fig. 2*D*). Worth mentioning is that the baseline level of Aβ plaques in the osmotic-pump PBS group was significantly lower than that of the IP PBS-treated control mice group (Fig. 2 *C*–*E*).

To further assess the observed changes in Aβ plaques, we investigated BACE-1 expression after NewStar2 administration. BACE-1 is responsible for cleaving APP, which will eventually lead to the formation and aggregation of toxic Aβ peptides (24). Mice treated with NewStar2 had reduced BACE-1 expression levels in the hippocampus compared with corresponding control mice irrespective of the mode of application (Fig. 2 *F* and *G*). In the corpus callosum area, mice treated IP with NewStar2 also had a decrease in BACE-1 levels compared with the control group. However, no significant differences were found in the osmotic pump groups with PBS versus NewStar2 application, although there was a tendency toward decreased BACE-1 expression in NewStar2-treated animals (Fig. 2*H*). No significant differences were found in the cortex area (Fig. 2*I*).

### Peripheral TNFR2 Stimulation by NewStar2 Expands Regulatory T Cells and Increases Activity of Glial Cells.

**Fig. 3.**
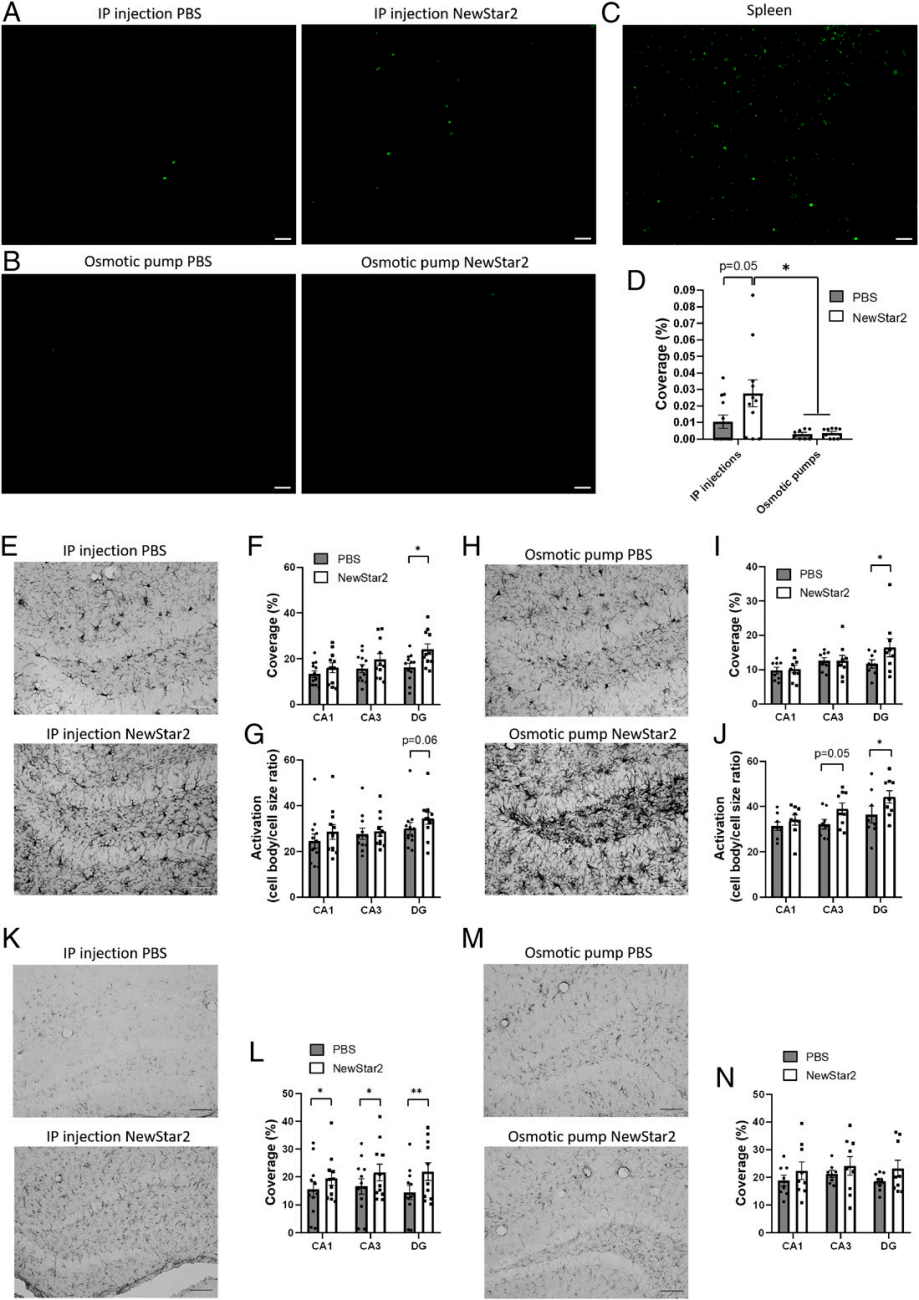
Tregs infiltration and microglial and astrocytic activation are increased after peripheral NewStar2 administration. (*A* and *B*) Hippocampal sections from mice treated with PBS or NewStar2 via IP injections or osmotic pumps were stained with Foxp3 (green) for Tregs. Representative images of hippocampus are shown. Scale bar, 50 µm. (*C*) Spleen sections, which regularly contain Tregs, were stained with Foxp3 (green) as a positive control. Scale bar, 50 µm. (*D*) Quantification of Foxp3 coverage in hippocampus (IP injections: PBS, *n* = 12, NewStar2, *n* = 11; osmotic pumps: PBS, *n* = 8, NewStar2, *n* = 9; two-way ANOVA, Bonferroni post hoc analysis: F_1,36_ = 2.91, degrees of freedom [DF] = 1). (*E* and *H*) IBA-1 microglia after PBS or NewStar2 administration via IP injections (*E*) or osmotic pumps (*H*). Representative images of DG are shown. Scale bar, 100 µm. (*F*) Quantification of microglia (IBA-1) coverage from CA1, CA3, and DG, shown in *E* (IP injections: PBS, *n* = 12, NewStar2, *n* = 11; two-way ANOVA, Bonferroni post hoc analysis: F_1,63_ = 8.72, DF = 1). (*G*) Quantification of microglial activation from CA1, CA3, and DG, shown in *E* (IP injections: PBS, *n* = 12, NewStar2, *n* = 11; two-way ANOVA, Bonferroni post hoc analysis: F_1,63_ = 2.05, DF = 1). (*I*) Quantification of microglia coverage from CA1, CA3, and DG, shown in *H* (osmotic pumps: PBS, *n* = 8, NewStar2, *n* = 9; two-way ANOVA, Bonferroni post hoc analysis: F_1,45_ = 6.24, DF = 1). (*J*) Quantification of microglial activation from CA1, CA3, and DG, shown in *I* (osmotic pumps: PBS, *n* = 8, NewStar2, *n* = 9; two-way ANOVA, Bonferroni post hoc analysis: F_1,45_ = 7.11, DF = 1). (*K* and *M*) GFAP astrocytes after PBS or NewStar2 administration via IP injections (*K*) or osmotic pumps (*M*). Representative images of DG are shown. Scale bar, 100 µm. (*L*) Quantification of astrocytes (GFAP) coverage from CA1, CA3, and DG, shown in *K* (IP injections: PBS, *n* = 12, NewStar2, *n* = 11; two-way ANOVA, Bonferroni post hoc analysis: F_1,63_ = 5.76, DF = 1). (*N*) Quantification of GFAP coverage from CA1, CA3, and DG, shown in *M* (osmotic pumps: PBS, *n* = 8, NewStar2, *n* = 9; two-way ANOVA, Bonferroni post hoc analysis: F_1,45_ = 3.03, DF = 1). Data are presented as mean ± SEM. *P < 0.05; **P < 0.01.

To determine the impact of NewStar2 on neuroinflammation, we first investigated in hippocampus sections the changes in the frequency of cells expressing forkhead box P3 (Foxp3), a specific marker for regulatory T cells (Tregs), a cell type which plays a key role in modulating immune responses (25). Mice treated IP with NewStar2 had a nearly significant (*P* = 0.05) threefold increase in Foxp3-positive Tregs in the hippocampus compared with PBS-treated control mice (Fig. 3 *A* and *D*). No significant differences were observed in the osmotic pump groups when comparing treatments, although both groups had significantly lower numbers of Foxp3-positive cells than did mice treated IP with NewStar2 (Fig. 3 *B* and *D*). Spleen sections were used as positive control (Fig. 3* C*).

Next, we examined the differences in the ionized calcium–binding adapter molecule 1 (Iba1) in cornu ammonis sector 1 (CA1), cornu ammonis sector (CA3), and dentate gyrus (DG) hippocampal areas after NewStar2 administration. Iba1 is a broadly used immunohistochemical marker for activated microglia and macrophages (26). We found that administration of NewStar2 via IP injections resulted in a significant increase in total Iba1 coverage in the DG area compared with IP PBS-treated mice (Fig. 3 *E* and *F*). Moreover, no significant changes were observed in microglial activation between IP NewStar2-treated and IP PBS-treated groups, although there was a tendency for increased microglial activation in the DG area of IP NewStar2-treated animals (Fig. 3*G*). Furthermore, we observed that central administration of NewStar2 via osmotic pumps caused a significant increase in total coverage (Fig. 3 *H* and *I*) and microglial activation (Fig. 3 *H* and *J*) in the DG area compared with corresponding controls. Microglial activation was also increased in the CA3 area, but the difference was not significant (Fig. 3*J*).

To evaluate the effect of NewStar2 on the inflammatory status of microglia, we investigated the changes in the purinergic receptor P2Y12 (P2RY12) in CA1, CA3, and DG hippocampal areas after NewStar2 administration. P2RY12 is expressed in microglia and platelets and has been identified as a specific marker for resting or nonactivated microglia, which enables the distinction between homeostatic (resting) and activated microglia (27, 28). We found that IP administration of NewStar2 led to a significant increase in P2RY12 total coverage in the CA3 and DG areas, but not in the CA1, compared with IP PBS-treated mice (*SI Appendix*, Fig. S3 *A* and *C*). In contrast, animals in the osmotic-pump groups did not show any significant differences when comparing treatments, although a tendency toward higher P2RY12 total coverage was observed in the CA3 and DG areas of NewStar2-treated animals (*SI Appendix*, Fig. S3 *B* and *D*).

Finally, to explore the effect of NewStar2 on astrocytic activity, we investigated the levels of glial fibrillary acidic protein (GFAP), an astrocyte activity marker. We found that IP administration of NewStar2 resulted in significantly higher GFAP total coverage in all areas of the hippocampus (CA1, CA3, and DG) compared with IP PBS-treated control mice (Fig. 3 *K* and *L*). However, central administration (i.e., via osmotic pump) of NewStar2 revealed no significant differences in GFAP total coverage compared with animals treated centrally with PBS, although a tendency toward increased GFAP coverage was observed in the NewStar2 treatment group (Fig. 3 *M* and *N*).

### NewStar2 Enhances Microglial Phagocytosis and Clearance of Aβ plaques.

**Fig. 4.**
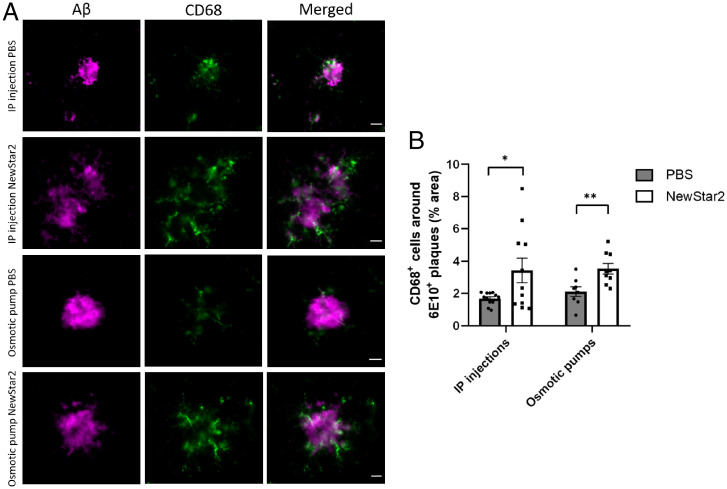
NewStar2 stimulates microglial phagocytosis and Aβ plaque uptake. (*A*) Sections from tissues of mice treated with PBS or NewStar2 via IP injections or osmotic pumps. Sections were stained with 6e10 (magenta) for Aβ plaques and CD68 (green) for phagocytic microglia. Representative images of the hippocampus area are shown. Scale bar, 25 µm. (*B*) Quantification of CD68-positive microglia around the border of Aβ plaques at a distance of 15 µm, shown in *A* (IP injections: PBS, *n* = 12 mice and *n* = 43 plaques, NewStar2, *n* = 11 mice and *n* = 29 plaques; osmotic pumps: PBS, *n* = 8 mice and *n* = 32 plaques, NewStar2, *n* = 9 and *n* = 24 plaques; two-way ANOVA, Bonferroni post hoc analysis: F_1,36_ = 11.35, degrees of freedom = 1). Data are presented as mean ± SEM. *P < 0.05; **P < 0.01.

To further investigate the effect of NewStar2 on microglia, we evaluated the association of Aβ plaques with CD68, a marker for phagocytic microglia. The presence of CD68-positive microglia in the vicinity of Aβ plaques has been related to clearance and degradation of Aβ plaques (29, 30). We observed a significant increase in CD68-positive microglia around Aβ plaques after administration of NewStar2 compared with controls in the IP and centrally treated mice compared with corresponding controls (Fig. 4 *A* and *B*).

### NewStar2 Is Neuroprotective and Crosses the Blood-Brain Barrier In Vitro.

The neuroprotective effect mediated by TNFR2 activation is dependent on the stimulation of the PI3K-PKB/Akt pathway (17, 31). Incubation of primary cortical neurons with increasing toxic concentrations of glutamate caused a dose-dependent decrease in PKB/Akt phosphorylation. This, however, was counteracted by pretreatment with 1 µg/mL NewStar2 for 24 h (*SI Appendix*, Fig. S4 *A*, *B* and *E*). Significantly higher PKB/Akt phosphorylation was observed in NewStar2-treated groups at 10, 25, and 50 µM glutamate concentrations compared with control groups (*SI Appendix*, Fig. S4*E*). Furthermore, to assess whether the neuroprotective effect of NewStar2 is glutamate dependent, we treated neurons with NewStar2 for 24 h, followed by incubation with a glutamate receptor antagonist (MK-801) prior to glutamate exposure. We observed that incubation with MK-801 prevented the decrease in PKB/Akt phosphorylation in the control groups (*SI Appendix*, Fig. S4 *C* and *F*). However, treatment with NewStar2 resulted in a significantly higher increase in PKB/Akt phosphorylation at the 25 and 50 µM glutamate concentrations (*SI Appendix*, Fig. S4 *D* and *F*). Overall, these results indicate that NewStar2 treatment has a neuroprotective effect in primary cortical neurons, which is not glutamate-receptor dependent.

Moreover, we tested whether different concentrations of NewStar2 labeled with fluorescein isothiocyanate (FITC) were able to cross the blood-brain barrier (BBB) in an in vitro transcytosis BBB model composed of human cerebral microvascular endothelial (hCMEC)/D3 cells. Addition of both 25 µg and 50 µg of FITC-NewStar2 showed a positive transcytosis passage of ∼3% across the BBB model (*SI Appendix*, Fig. S5).

## Discussion

It has been widely accepted that TNF-α plays a key role in mediating the neuroinflammatory response in AD. However, anti–TNF-α therapies have largely failed in the treatment of neurodegenerative diseases, probably due to the antithetic functions of the two TNF receptors, with TNFR1 being responsible for proinflammatory and proapoptotic signaling, whereas TNFR2 signaling induces immunomodulatory and regenerative processes. Moreover, the exact effects of triggering TNFR2 in the AD context remain unknown. In the present study, we hypothesized that selective stimulation of TNFR2 could attenuate the Aβ-induced cognitive impairment and the AD-related neuropathology. We demonstrated that exogenous therapeutic activation of TNFR2 drastically reduces Aβ plaque load and rescues AD-related cognitive impairments in the J20 mouse model.

Upon TNFR2-agonist treatment, we found a clear reduction in Aβ plaque load in specific areas of the brain that are affected in AD, such as the hippocampus and cortex. The significant reduction in Aβ deposition via TNFR2 agonist was accompanied by significantly lower levels of BACE-1. BACE-1 is a major player in Aβ formation since it contributes to the cleavage of APP into Aβ_42_ (32). It is well known that activation of TNFR2 and its downstream PI3K/Akt pathway is related to neuroprotection and immune regulation (9). Indeed, it has been demonstrated that activation of the PI3K/Akt pathway resulted in the inhibition of BACE-1 expression in the APP/PS1 AD mouse model (33). Thus, our results suggest that activation of TNFR2 by NewStar2 leads to the up-regulation of the PI3K/Akt pathway and reduction in BACE-1 levels, which, in turn, results in decreased toxic Aβ peptide formation. Other studies have also investigated the levels of BACE-1 after administering AD-targeted therapies. For instance, intragastric administration of icaritin (a flavonoid derivative) resulted in an improvement in spatial memory and a reduction in Aβ and BACE-1 expression in the SAMP8 mouse model (34). Moreover, intrahippocampal injection of agmatine (a biogenic amine related to neuroprotection) was able to rescue memory deficits in an acute-AD mouse model induced by intracerebroventricular injection of Aβ_1–42_, although no differences were observed in APP or BACE-1 expression (35). In addition, several studies have focused on inhibiting BACE-1 as therapy to avoid the production of toxic Aβ peptides. However, the vast majority of clinical trials with BACE-1 inhibitors had to be discontinued due to various adverse effects such as worsening of cognitive functions, psychiatric events, or liver toxicity (36–39). TNFR2 agonists, however, may be an alternative treatment strategy, since preliminary findings showed that they are well tolerated and safe in preclinical models (17, 40).

We next evaluated microglia as potential players in influencing plaque load. Microglia are the resident macrophages in the CNS and their function is to maintain homeostasis and react against pathogens. It has been demonstrated that microglia activation is necessary for Aβ clearance in AD mouse models (20); however, constant activation and release of proinflammatory cytokines can inhibit the phagocytic activity of microglia and lead to chronic neuroinflammation (23, 41). Importantly, TNF-α signaling and microglial activation–related genes have been identified as genetic risk factors in AD pathophysiological processes in a recent, in-depth genome-wide association study (42). Thus, in the present study, we investigated and characterized the effect of TNFR2 engagement on different microglial markers. First, when examining Iba1, a marker of activated microglia, we found that NewStar2 treatment led to an increase in microglia activation and coverage compared with controls. The increase in microglial activation was more prominent in the osmotic-pump groups, possibly due to the inflammatory reaction caused after the cannula implantation in the brain. Nevertheless, microglial activation in mice treated with NewStar2 centrally via osmotic pumps was significantly higher than the corresponding PBS group, meaning that despite the inflammatory reaction, NewStar2 further contributed to the increase in microglial activation. To further validate the role of microglia in this study, we investigated the levels of CD68, a marker of phagocytic microglia, in relation to Aβ plaques. We observed that administration of NewStar2, both centrally and IP, led to an increase in CD68-positive microglia cells around Aβ plaques. These results imply that activation of TNFR2 leads to an increase in the phagocytic activity of microglia, which probably results in a higher Aβ clearance and, thus, an eventual reduction in plaque load. In support of this hypothesis, a study by Gao et al. (43) showed that TNFR2-deficient microglia developed a proinflammatory phenotype and showed reduced phagocytic activity in the experimental autoimmune encephalomyelitis model. Moreover, it has been demonstrated that TNFR2 agonists expand Tregs (40, 44), which have been proposed to play a beneficial role in AD by promoting a type 1 interferon–dependent protective activation profile in microglia (45). Indeed, we found in our study that there was a threefold increase in Foxp3-positive Tregs in the brain of IP NewStar2-injected mice compared with PBS-treated control mice. In support of our results, a study performed in APP/PS1 mice showed that expansion of Tregs led to improved cognitive functions and an increased number of plaque-associated microglia (45). Findings from that study and our own data suggest that peripheral injection of NewStar2 promotes Tregs expansion and infiltration into the CNS, which may enhance microglial phagocytic activity. Other studies have also demonstrated the role of microglia in AD mouse models. For instance, stereotaxic injection of soluble triggering receptor expressed on myeloid cells 2 resulted in a decrease in Aβ plaques, improvement in spatial memory, and increased microglial phagocytic activity in the 5xFAD AD mouse model (46). Additionally, subcutaneous injection of hematopoietic growth factors such as stem cell factor and granulocyte colony-stimulating factor led to a reduction in Aβ plaques and increase in microglial markers Iba1 and CD68 around Aβ plaques in the APP/PS1 AD mouse model (30).

Several studies have related AD and microglial activation with a decrease in the marker P2RY12, initially described as a marker for homeostatic/nonactivated microglia (28, 47, 48). Moreover, when analyzing different AD cases, a study found an absence of P2RY12-positive microglia around Aβ plaques or areas related to inflammation (49). In the present study, we found that activation of TNFR2 with NewStar2 increased P2RY12 levels overall in different areas of the hippocampus. Because we observed an increase in both microglial activation (Iba-1, CD68) and nonactivated microglia markers (P2RY12) after NewStar2 administration, our results contradict the aforementioned studies in which an increase in activated microglia was correlated with decreased levels of P2RY12. However, a recent study suggested that P2RY12 could be expressed not only in nonactivated microglia but also in a wider variety of microglial phenotypes. Indeed, P2RY12-positive microglia were found near diffuse Aβ plaques and colocalizing with CD68-positive microglia (50). Moreover, the presence of P2RY12-positive microglia has been suggested to be protective against AD neuropathology (51). Thus, the homeostatic/proinflammatory role of P2RY12-positive microglia in the AD context remains unclear.

Similarly to microglia, activated astrocytes are found near Aβ plaques and are partly responsible for the neuroinflammatory processes in AD (52, 53). Nevertheless, active astrocytes have been suggested to play a role in Aβ degradation (54). We found that activation of TNFR2 by NewStar2 resulted in a significant increase in active astrocytes coverage in different areas of the hippocampus when comparing IP NewStar2-treated and IP PBS-treated groups. In contrast, we observed no significant differences when comparing both osmotic-pump groups, although the coverage in both groups was comparable to that of the IP NewStar2-treated group. These results suggest that the increased astrogliosis observed in the IP NewStar2-treated group and both osmotic-pump groups could contribute to Aβ clearance and, thus, to the observed reduction in Aβ plaques. However, a study performed in animal models of chronic stress showed that an increase in reactive astrocytes could be related to an increase in Aβ, since astrocytes overexpress BACE-1 under chronic stress conditions (55). Our study findings contradict those of Rossner et al. (55) since we observed an overall increase in reactive astrocytes accompanied by a reduction in BACE-1 levels.

At the behavioral level, we showed that NewStar2 did not affect body weight or anxiety levels. Evaluation of working memory in the Y-maze test revealed that NewStar2 improved working memory in the IP NewStar2-treated group compared with the IP PBS-treated control group. However, no improvement was found when comparing both osmotic-pump groups. Besides, we demonstrated that NewStar2 was able to improve spatial memory in the MWM test when comparing IP NewStar2-treated and IP PBS-treated groups. Interestingly, we did not observe any differences between the osmotic-pump groups. However, the escape latency, platform crossings, and time spent in the platform quadrant in both groups were similar to those of IP NewStar2-treated mice, meaning that both osmotic-pump groups showed an improved spatial memory. This distribution pattern was also observed in the results of the Aβ plaques from the present study: both osmotic-pump groups and IP NewStar2-treated mice presented significantly lower Aβ deposition compared with the IP PBS-treated control group. Furthermore, overall Iba1 and GFAP activation was also increased in both osmotic-pump groups and the IP NewStar2-treated group compared with the IP PBS-treated control group. These findings suggest that the sole implantation of a cannula in the brain that infuses PBS for a period of 6 wk would cause an inflammatory reaction that may serve as strategy to ameliorate plaque deposition and rescue cognitive functions. This inflammatory reaction caused by the cannula implantation in the brain might be responsible for the observed increase in the activation of glial cells, which could, in turn, result in the relative reduction in Aβ plaques and improved cognition in the centrally treated PBS group. Nevertheless, activation of TNFR2 by central administration of NewStar2 further improved cognitive functions and ameliorated AD-related pathology compared with central infusion of PBS. This outcome would potentially open the possibility of administering an AD therapy via IP injections instead of centrally, which would involve fewer risks and a less invasive and more patient-friendly treatment. Importantly, we can conclude that, based on our results, peripheral administration of NewStar2 is equivalent or even more effective in attenuating AD pathology than is central administration. Future research should, therefore, also focus on the involvement and mechanistic functions of peripheral immune cells on the pathophysiology of AD.

Other TNF-related therapies have been tested in animal models of AD. For example, a study performed in the J20 AD mouse model showed that inhibition of lipocalin-2, a proinflammatory protein released upon TNFR1 activation, failed to improve cognitive functions and did not affect plaque load or glia activation (56). In contrast, a study in 5xFAD mice using XPro-1595, an inhibitor of sTNF, resulted in a reduction in Aβ plaques in the subiculum and an improvement in long-term potentiation (15). Moreover, in an acute neurodegenerative mouse model, administration of a combination of a TNFR1 antagonist (ATROSAB) and a TNFR2 agonist (EHD2-scTNFR2) was protective against excitotoxicity and rescued cognitive impairments (17). Furthermore, in vitro studies have also validated the protective functions of stimulating TNFR2. For instance, the TNFR2 agonist TNC-scTNFR2 was protective against neuronal cell death caused by oxidative stress (16). Moreover, activation of TNFR2 protected primary neurons from excitotoxicity and activated the PI3K-PKB/Akt prosurvival pathway (31). That study’s findings are in line with the results from the present study, in which preincubation of primary cortical neurons with NewStar2 was neuroprotective, based on a significant increase in PKB/Akt phosphorylation. In addition, we showed that ∼3% of NewStar2 was able to cross the BBB in an in vitro BBB model. In fact, it has previously been demonstrated that TNF-α is transported across the BBB via TNFR1 and TNFR2 (57, 58). Even though the passage of NewStar2 across the BBB has only been tested in an in vitro model so far, this result suggests that, due to its similar structure to TNF-α and receptor binding, NewStar2 could possibly cross the BBB in vivo via the same mechanisms, although this remains to be determined. In summary, previous studies and the results provided by the present investigation imply that TNFR2 can successfully be used as potential therapeutic target for AD.

Limitations of this study should be considered for the interpretation of our findings. First is the employment of the J20 mouse model. Although neuroinflammation and Aβ plaques are present in this animal model of AD, it lacks the presence of neurofibrillary tangles, an important hallmark of AD. This means that translation of the effect of NewStar2 to humans requires further investigation.

In this in vivo study, we investigated the potential effects of selective targeting TNFR2 in a model of AD. We demonstrated that administration of NewStar2 ameliorates AD neuropathology and rescues cognitive impairments. Although the exact mechanism by which NewStar2 mitigates AD neuropathology remains to be determined, we speculate that NewStar2 is able to drastically reduce plaque deposition through a combination of decreased Aβ production due to lower BACE-1 levels and increased Aβ clearance due to higher astrocytic and microglial phagocytic activity. We further hypothesize that the increased phagocytosis and clearance rate of glial cells observed after peripheral administration of NewStar2 might be induced by a combination of Tregs expansion and migration to the CNS together with the potential penetration of NewStar2 in the brain across the BBB. Moreover, this study has proven that the sole implantation of a cannula in the brain of mice exerts an inflammatory reaction that can mitigate, to some extent, AD pathology. However, central administration of NewStar2 further ameliorated AD pathology.

In conclusion, our data provide strong evidence that TNFR2 stimulation is effective in ameliorating Aβ deposition and AD-related cognitive impairments and in modulating the phagocytic activity of glial cells in the CNS. Even though some of the effects of NewStar2 need additional investigation, our study sheds light on the effects of stimulating TNFR2 in AD by demonstrating a direct neuroprotective role of TNFR2 activation in attenuating AD pathology.

## Materials and Methods

### TNFR2 Stimulation.

To selectively engage TNFR2, we used a new TNFR2 agonist, designated as NewStar2 or irrIgG1(N297A)-HC:sc(mu)TNF80 (59), which is based on Star2 [TNC-sc(mu)TNF80], a fusion protein of the trimerization domain of tenascin-C and three peptide-linker-connected (thus single-chain (sc) encoded) murine TNF (muTNF) protomers with mutations conferring specificity for TNFR2 (40). To generate NewStar2, the TNFR2-specific, sc–encoded sc(mu)TNF80 domain of Star2 was fused to the C terminus of the heavy chain of a human IgG1 with a mutation (N297A) avoiding interaction with FcγRs and irrelevant specificity in mice. Thus, while Star2 comprises, in total, nine TNFR2-specific TNF protomers, NewStar2 only comprises six TNFR2-specific TNF protomers. Irrespective of this difference, however, both variants are in vitro comparably active. Since IgG1(N297A) still interacts with the neonatal Fc receptor, NewStar2 [irrIgG1(N297A)-HC:sc(muTNF80)], however, has enhanced serum retention and, thus, much better in vivo activity than Star2. NewStar2 is produced by transient transfection of Hek293 cells with expression plasmids encoding the light chain and the sc(mu)TNF80 fusion protein of the heavy chain of the irrelevant IgG1(N297A) (59). NewStar2 was purified by affinity purification on anti-Flag agarose by help of internal Flag tags present in the antibody. Transient transfection and anti-Flag agarose purification were performed essentially as described elsewhere for scFv fusion proteins (60).

### Cytotoxicity Assay on Kym-1 Cells.

To test the stability of NewStar2, a cytotoxicity assay using crystal violet was performed in Kym-1 cells. NewStar2 was placed in an incubator for 6 wk at 37 °C and its bioactivity was compared with that of NewStar2 stored at 4 °C.

In brief, Kym-1 cells were cultivated in RPMI 1640 medium supplemented with 10% fetal bovine serum (FBS), 5% penicillin/streptomycin, and 5% l-glutamine. Kym-1 cells (1.5 × 10^4^ cells/well) were transferred to 96-well cell-culture plates and treated with 500 ng/mL NewStar2 for 24 h at 37 °C. Cells were washed with PBS and incubated with crystal violet (0.5% crystal violet, 20% methanol) for 20 min. Crystal violet was removed by intensively rinsing with water and cells were air-dried overnight. Methanol was added to resolve the crystal violet and optical density at 550 nm was measured. Each sample was analyzed in six replicates, using Microsoft Excel and IBM SPSS Statistics 26.0.

### Primary Cortical Neuron Culture.

Cortical neurons were treated with NewStar2 to assess the potential neuroprotective effect of TNFR2 activation on glutamate-induced neuronal cell death. Primary cortical neurons were prepared as previously described (31). Primary cortical neurons were obtained from brain embryos of wild-type pregnant mice at embryonic day 14. Meninges and olfactory bulbs were carefully detached from the brains, and cortical neurons were separated by mechanical dissociation. Neurons (6 × 10^4^ cells/well) were plated in a 96-well polyethyleneimine-precoated plate. Primary neurons were cultured using neurobasal medium (Life Technologies) supplemented with 2% B-27 supplement, 0.2% penicillin/streptomycin, and 0.25% l-glutamine (Life Technologies). After 48 h, 10 µM cytosine arabinoside (Sigma) was added to the cells for 24 h to prevent nonneuronal cell growth, and the medium was subsequently completely refreshed. After 7 d of culture, neurons were preincubated with 1 µg/mL NewStar2 for 6 or 24 h and were subsequently treated with different concentrations of glutamate (0, 5, 10, 25, or 50 µM; Sigma) for 1 h. When necessary for the experiment, cells were incubated with a glutamate-receptor antagonist, MK-801 (Sigma), for 1 h prior to glutamate exposure. Then, the medium was refreshed, and neurons were allowed to recover for 24 h. Next, cells were collected for Western blot analyses.

### Western Blot Analysis (PI3K-PKB/Akt).

The neuroprotective effects of TNFR2 stimulation were evaluated by measuring protein expression levels of PKB/Akt. Cells were washed twice with PBS and directly lysed by addition of 250 µL lysis buffer (50 mM Tris⋅HCl, pH 7.4; 1% Nonidet P-40; 0.25% sodium deoxycholate; 150 mM NaCl; 1 mM ethylenediaminetetraacetic acid; complete mini protease-inhibitor mixture tablet). Cell lysates were centrifuged at 4 °C (30 min at 9,600*g*), and the protein concentration of supernatants was measured by the Bradford assay and adjusted to 1 µg/µL. Twenty micrograms of total protein were denatured in Laemmli buffer for 10 min at 70 °C and separated by 4–12% sodium dodecyl sulfate–polyacrylamide gel electrophoresis (160V, 40 min). Proteins were then transferred to polyvinylidene difluoride membranes and blocked with 5% skim-milk powder solution in Tris-buffered saline (TBS) containing 0.1% Tween 20 for 1 h. Subsequently, membranes were incubated overnight at 4 °C with primary antibodies of the specificity of interest: anti–phospho-Akt Ser-473 (1:500; #9271, Cell Signaling); anti-Akt (1:2,000; #9272, Cell Signaling). After that, membranes were washed with TBS containing 0.1% Tween 20 and incubated with horseradish peroxidase–conjugated secondary antibody goat anti-rabbit (1:10.000; #7074, Cell Signaling) in TBS containing 0.1% Tween 20 for 2 h at room temperature. Immunoreactivity was detected using enhanced chemiluminescence (Pierce Biotechnology) with a ChemiDoc XRS system (Bio-Rad Laboratories). GAPDH expression was analyzed as loading control.

### Mice.

To investigate the effects of NewStar2 at the behavioral and pathological levels, the J20 AD mouse model was used. J20 mice overexpress human APP with two mutations (Swedish and Indiana), and Aβ plaques can be observed in the brain between 5 and 7 mo of age (61). J20 mice were initially obtained from the Mutant Mouse Resource and Research Center (stock no. 034836-JAX; former JAX stock no. 006293) and were thereafter bred in our animal facility on a C57BL/6 background. J20 mice were hemizygous for the J20 transgene, which was verified by PCR using appropriate primers (*SI Appendix*, Table S1). Mice had access to food and water ad libitum and were on a 12:12 light/dark cycle. All animal experiments performed in this study were approved by the animal ethics committee of the University of Groningen (approval no. AVD1050020186146).

### NewStar2 Administration.

NewStar2 or PBS as a control was administered to a total of 49 male mice either centrally via the implantation of mini-osmotic pumps connected to an intracerebroventricular cannula (NewStar2: *n* = 12; PBS: *n* = 11) or systemically via IP injections (NewStar2: *n* = 13; PBS: *n* = 13). The treatment started at 6 mo of age and lasted for 6 wk. After that, animals were subjected to different behavioral tests and, finally, mice were euthanized to collect brain tissue, blood, and cerebrospinal fluid (Fig. 1*A*).

### Central Administration Via Implantation of Mini-Osmotic Pumps.

NewStar2 was administered centrally via the subcutaneous implantation of a mini-osmotic pump (0.15 μL/h delivery rate for 6 wk; Model 2006, Alzet) connected to a cannula into the lateral ventricle, which allows constant infusion of the compound. Pumps delivered either PBS or NewStar2 (10 µg/wk) dissolved in PBS. Before the surgical procedure, pumps were filled with the corresponding solution and incubated in PBS at 37 °C for at least 60 h to ensure immediate pumping at the moment of implantation. Mice were anesthetized with 5% isoflurane and fixed in a Kopf stereotactic frame (Kopf Instruments Model 900). The body temperature of the mice was kept at 37 °C, using a heating blanket, during the whole surgical procedure. One small hole was drilled into the skull for the placement of the cannula in the lateral ventricle (coordinates: anteroposterior,0.05 mm; lateral, 0.1 mm; dorsoventral, 0.25 mm), and a second hole was drilled for the placement of an anchoring screw. A subcutaneous pocket was made in the back of the animal to accommodate the pump. The osmotic pump was inserted in the subcutaneous pocket and the cannula fixed on a cannula holder. The cannula was slowly introduced into the first hole at a depth of 2.5 mm. Subsequently, dental cement was added to the cannula, as was an anchoring screw, to properly fix the cannula to the skull. After the dental cement dried, the incision was sutured and mice were returned to their cage. Mice were monitored until total recovery from anesthesia.

### Systemic Administration via Peripheral Injections.

NewStar2 (2.5 mg/kg in 200 µL of PBS) or PBS were administered systemically twice a week for a period of 6 wk via IP injections.

### Behavioral Tests.

After the completion of the 6-wk treatment, mice were subjected to different behavioral tests to investigate their cognitive function. Tests were performed in the following order: EPM, Y-maze spontaneous alternation, and MWM to minimize the effects of stressful events on subsequent testing procedures. All behavioral tests were performed in a separate experimental room and were conducted by the same experimenter, who was blinded to treatments.

### Habituation.

Prior to the start of the behavioral tests, mice were habituated to handling by the researcher and to the experimental room. Each mouse was separately handled in the experimental room for 2 min during 5 consecutive days before the start of the behavioral tests.

### EPM.

To evaluate anxiety-like behavior, mice were tested in an EPM (62). The maze was elevated 60 cm from the ground and consisted of four arms (5.5 cm wide × 30 cm long) positioned in a plus shape: two opposite open arms and two closed arms with an open center area in the middle connecting the four arms. Light intensity was set to ∼10 lx in the center of the maze and ∼12 lx in the open arms. During the test, mice were placed in the center of the maze and were allowed to freely explore the maze for 8 min. The percentage of time spent in the center and the open and closed arms, as well as the number of arm entries and total number of entries, were determined using EthoVision XT system.

### Y-Maze Spontaneous Alternation.

To assess spatial working memory, mice were tested in a Y maze to measure spontaneous arm alternation (63). The maze consisted of three arms (8 cm wide × 40 cm long) separated at a 120° angle from each other. Light intensity was set to ∼10 lx in the center of the maze. Mice were placed in the middle of the maze and were allowed to freely explore the three arms for 10 min. The percentage of spontaneous alternation was calculated manually based on the number of triads [i.e., number of triads/(total number of entries − 2) × 100]. Triads were described as three consecutive entries into all three different arms.

### MWM.

To investigate hippocampus-dependent spatial memory, mice were tested in the MWM (64). Mice were tested in a circular pool (135-cm diameter). Water-based nontoxic white paint (Dullux Roll-it Easy) was added to the water to make the platform imperceptible to the mice. Water temperature was maintained at 24 °C throughout the test. Various visual cues were placed on the walls of the experimental room, and the light intensity was ∼40 lx in the center of the maze. The test consisted of a training phase and a probe trial phase. During the training phase, mice were trained for 8 consecutive days to learn the fixed location of a platform (15-cm diameter), hidden 1.5 cm under the water. Each day consisted of a training block of four trials with a maximum of 120 s swimming per trial per mouse. Escape latency, swimming distance, and swimming speed were scored using the EthoVision XT system. Following the training phase, the probe trial phase was conducted for 2 consecutive days (24 and 48 h after the last day of the training phase). To this end, the platform was removed from the maze and mice were allowed to freely swim once for 100 s. The number of crossings through the platform location and time spent in the different quadrants of the pool were determined using EthoVision XT system.

### Perfusion and Postfixation.

After the last behavioral test was completed, mice were anesthetized with sodium pentobarbital and transcardially perfused with 0.9% heparin saline and 4% paraformaldehyde. Brains, blood and cerebrospinal fluid were collected from each animal. Brains were postfixed for 24 h in 4% paraformaldehyde and then extensively washed with 0.01 M phosphate buffer, after which brains were cryoprotected and dehydrated with 30% sucrose, frozen, and cut in 20 μm–thick coronal sections using a cryostat at −20 °C.

### Immunohistochemistry for Detection of Aβ, Iba1, GFAP, and P2RY12.

To assess the neuropathological effects of NewStar2, several immunohistochemical analyses were completed. All procedures were performed on hippocampal free-floating sections. Sections were washed 3 × 5 min in 0.01 M TBS and then incubated in 0.3% hydrogen peroxide (H_2_O_2_) in 0.01 M TBS for 30 min to inactivate endogenous peroxidases. Sections were washed 3 × 5 min in 0.01 M TBS, preincubated with blocking buffer (3% BSA, 0.1% Triton X-100 in 0.01 M TBS) for 1 h and incubated with primary antibodies (in blocking buffer) at 4 °C for 48 h (anti-Aβ) or 72 h (anti–Iba-1, anti-GFAP, anti-P2RY12). Next, sections were washed 6 × 5 min in 0.01 M TBS and incubated with Biotin-SP–conjugated secondary antibodies (anti-mouse, 1:500 [#115–065-166]; anti-rabbit, 1:500 [#111–065-045]; Jackson ImmunoResearch Laboratories) for 2 h. After washing 6 × 5 min in 0.01 M TBS, sections were incubated in avidin-biotin complex (1:500; Vectastain Standard ABC kit, Vector Laboratories) for 1 h. Subsequently, the staining was developed using 3,3′-diaminobenzidine (DAB) at a concentration of 0.7 mg/mL (SigmaFAST DAB tablets, Sigma). The DAB reaction was initiated by the addition of 0.0033% H_2_O_2_ and stopped by three quick washes in 0.01 M TBS. Sections were left in TBS overnight at 4 °C. Afterward, sections were mounted onto Menzel Superfrost glass microscope slides (Thermo Scientific), using 1% gelatin, and left to dry overnight. Next, mounted sections were dehydrated through an ethanol to xylol series (2 × 5 min 100% ethanol; 1 × 5 min 70% ethanol/30% xylol; 1 × 5 min 30% ethanol/70% xylol; 3 × 5 min 100% xylol) and coverslipped using DPX mounting medium (Sigma). The following primary antibodies were used: mouse anti-Aβ_1–16_ (1:2,000; 6e10, BioLegend), mouse anti-GFAP (1:10000; #G3893, Sigma), rabbit anti-Iba1 (1:2,500; #019–19741, Wako Chemicals), and rabbit anti-P2RY12 (1:1,000; #P4871, Sigma). Images were taken using an Olympus BH2 microscope (Leica QWin Software) at a ×100 (Aβ, GFAP), ×200 (Iba1) or ×400 (P2RY12) magnification.

### Immunohistochemistry for detection of Aβ + BACE-1, Aβ + CD68, and Foxp3 fluorescent stainings.

Free-floating hippocampal sections (20 μm) were washed 3 × 5 min in 0.01 M TBS, preincubated with blocking solution (3% BSA, 0.1% Triton X-100 in 0.01 M TBS) for 1 h, and incubated with primary antibodies (in blocking buffer) at 4 °C for 24 h. Next, sections were washed 6 × 5 min in 0.01 M TBS and incubated with corresponding Alexa Fluor–conjugated secondary antibodies (AF488-conjugated donkey anti-rabbit, 1:500 [#A21206]; AF555-conjugated donkey anti-mouse, 1:500 [#A32773]; and AF488-conjugated donkey anti-rat, 1:500 [#A21208]; Invitrogen) for 2 h in the dark. Subsequently, sections were washed 3 × 5 min in 0.01 M TBS, mounted onto Menzel Superfrost glass microscope slides (Thermo Scientific) in TBS, and coverslipped using Mowiol (Sigma) as a mounting medium. The following primary antibodies were used: mouse anti-Aβ_1–16_ (1:2,000; 6e10, BioLegend), rabbit anti-BACE-1 (1:200; #5606, Cell Signaling), rat anti-CD68 (1:1,000; #MCA1957GA, Bio-Rad), and rat anti-Foxp3 (1:500; #14–5773-82, eBioscience). Fluorescent images were obtained using the Leica DMI6000 B microscope (Leica Microsystems) at a ×400 magnification (Aβ + CD68) or ×200 (Aβ + BACE-1 and Foxp3).

### Quantification of immunohistochemical stainings.

For quantification of Aβ, CD68, GFAP, BACE-1, and P2RY12, ×100 or ×400 magnification images were analyzed using ImageJ. The percentage of coverage was quantified by automatically dividing positively stained structures from the selected regions of interest (hippocampus, cortex, corpus callosum) by the area of the image field. For quantification of Iba1, both the percentages of coverage and microglial activation were determined in ×200-magnification images. Microglial activation was quantified based on the protocol by Hovens et al. (65) by calculating cell body to cell size ratio. Specific hippocampal regions (CA1, CA3, and DG) were included in the Iba1, GFAP, and P2RY12 analysis.

### FITC labeling of NewStar2.

NewStar2 was brought to a sodium bicarbonate buffer (pH 9) at a protein concentration of 2 mg/mL using a 10-kDa molecular weight–cutoff dialysis cassette (Thermo Scientific #66380) with three buffer changes over a 24-h period. FITC (Sigma Aldrich #7250) was dissolved in dimethyl sulfoxide at a concentration of 2 mg/mL; 2 mg of NewStar2 and 30 µg of FITC were mixed and incubated for 2 h at room temperature. Unbound FITC was removed by dialysis against PBS (pH 7.4) with three buffer changes over a 24-h period using a 10-kDa molecular weight–cutoff dialysis cassette (Thermo Scientific #66380).

### FITC-NewStar2 transcytosis assays.

As previously described (66), hCMEC/D3 cells were maintained in 25-cm^2^ flasks precoated with 150 μg/mL rat-tail collagen type-I (Enzo LifeSciences) in endothelial basal medium (EBM) 2 (Lonza) supplemented with 1 ng/mL human basic fibroblast growth factor (Peprotech), 5 μg/mL ascorbic acid (Sigma-Aldrich), 1.4 μM hydrocortisone (Sigma-Aldrich), 10 mM HEPES (Gibco), 1% (volume per volume) chemically defined lipid concentrate (Gibco), 5% (volume per volume) FBS, 100 units/mL penicillin and 100 μg/mL streptomycin at 37 °C in a humidified atmosphere with 5% CO_2_. hCMEC/D3 cells were plated at 50,000 cells/cm^2^ on 1-μm pore–size polycarbonate membrane Transwell inserts (Corning) and allowed to grow until complete confluency within 5 d. Transwell inserts were coated with 150 μg/mL rat-tail collagen type I. Medium was changed every 48 h. On day 5, cells were washed with prewarmed Hanks' Balanced Salt Solution (HBSS), and 25 μg or 50 μg FITC-NewStar2 was added apically in EBM + 5% FBS, and incubated for 4 h at 37 °C. We added 1,000 μL EBM + 5% FBS medium basally. Apical and basal media were collected in labeled Eppendorf tubes after 4 h. Then, filters were washed once with 500 μL of HBSS, which was added to the apically collected 500 μL of medium. Subsequently, filters were cut out and soaked in 500 μL fH_2_O for 10 min to osmotically burst cells, and 500 μL EBM + 5% FBS medium was added to the solution. The fluorescence intensity in apical, cellular, and basal media was measured using a FluoStar Optima plate reader (BMG Labtech). Background fluorescence was subtracted from each reading before the relative signal intensity was calculated for each compartment. Relative fluorescence per compartment was calculated by dividing the fluorescence per compartment by the sum fluorescence per well.

### Statistics.

Results were analyzed by unpaired Student’s *t* test when comparing two normally distributed groups or Mann-Whitney test when data were not normally distributed. One-way ANOVA followed by Tukey’s multiple comparison post hoc test or two-way ANOVA followed by Bonferroni multiple-comparison post hoc test were used to analyze multiple groups. All statistical tests performed were two-tailed. All data are reported as mean ± SEM and statistically significant differences were considered when *P* < 0.05. Statistical analyses were performed using GraphPad Prism 8.0 for Windows.

## Supplementary Material

Supplementary File

## Data Availability

All study data are included in the article and/or supporting information.

## References

[1] R. Fischer, O. Maier, Interrelation of oxidative stress and inflammation in neurodegenerative disease: Role of TNF. Oxid. Med. Cell. Longev. 2015, 610813 (2015). 25834699 10.1155/2015/610813 PMC4365363

[2] F. Brosseron, M. Krauthausen, M. Kummer, M. T. Heneka, Body fluid cytokine levels in mild cognitive impairment and Alzheimer’s disease: A comparative overview. Mol. Neurobiol. 50, 534–544 (2014). 24567119 10.1007/s12035-014-8657-1 PMC4182618

[3] W. Swardfager , A meta-analysis of cytokines in Alzheimer’s disease. Biol. Psychiatry 68, 930–941 (2010). 20692646 10.1016/j.biopsych.2010.06.012

[4] N. Ortí-Casañ , Targeting TNFR2 as a novel therapeutic strategy for Alzheimer’s disease. Front. Neurosci. 13, 49 (2019). 30778285 10.3389/fnins.2019.00049 PMC6369349

[5] O. V. Atigari, D. Healy, Schizophrenia-like disorder associated with etanercept treatment. BMJ Case Rep. 2014, bcr2013200464 (2014). 10.1136/bcr-2013-200464 PMC3902342 24419811

[6] J. Butchart , Etanercept in Alzheimer disease: A randomized, placebo-controlled, double-blind, phase 2 trial. Neurology 84, 2161–2168 (2015). 25934853 10.1212/WNL.0000000000001617 PMC4451045

[7] N. L. Sicotte, R. R. Voskuhl, Onset of multiple sclerosis associated with anti-TNF therapy. Neurology 57, 1885–1888 (2001). 11723281 10.1212/wnl.57.10.1885

[8] The Lenercept Multiple Sclerosis Study Group and The University of British Columbia MS/MRI Analysis Group, TNF neutralization in MS: Results of a randomized, placebo-controlled multicenter study. Neurology 53, 457–465 (1999). 10449104

[9] Y. Dong, D. Dekens, P. P. De Deyn, P. Naudé, U. Eisel, Targeting of tumor necrosis factor alpha receptors as a therapeutic strategy for neurodegenerative disorders. Antibodies (Basel) 4, 368–408 (2015).

[10] R. Fischer, R. Kontermann, O. Maier, Targeting sTNF/TNFR1 signaling as a new therapeutic strategy. Antibodies (Basel) 4, 48–70 (2015).

[11] S. K. Williams , Anti-TNFR1 targeting in humanized mice ameliorates disease in a model of multiple sclerosis. Sci. Rep. 8, 13628 (2018). 30206422 10.1038/s41598-018-31957-7 PMC6133964

[12] P. M. Madsen , Oligodendroglial TNFR2 mediates membrane TNF-dependent repair in experimental autoimmune encephalomyelitis by promoting oligodendrocyte differentiation and remyelination. J. Neurosci. 36, 5128–5143 (2016). 27147664 10.1523/JNEUROSCI.0211-16.2016 PMC4854972

[13] C. J. Barnum , Peripheral administration of the selective inhibitor of soluble tumor necrosis factor (TNF) XPro1595 attenuates nigral cell loss and glial activation in 6-OHDA hemiparkinsonian rats. J Park Dis 1, 349–360 (2014). 10.3233/JPD-140410 PMC4154985 25061061

[14] C. Cavanagh , Inhibiting tumor necrosis factor-α before amyloidosis prevents synaptic deficits in an Alzheimer’s disease model. Neurobiol. Aging 47, 41–49 (2016). 27552480 10.1016/j.neurobiolaging.2016.07.009

[15] K. P. MacPherson , Peripheral administration of the soluble TNF inhibitor XPro1595 modifies brain immune cell profiles, decreases beta-amyloid plaque load, and rescues impaired long-term potentiation in 5xFAD mice. Neurobiol. Dis. 102, 81–95 (2017). 28237313 10.1016/j.nbd.2017.02.010 PMC5464789

[16] R. Fischer , A TNF receptor 2 selective agonist rescues human neurons from oxidative stress-induced cell death. PLoS One 6, e27621 (2011). 22110694 10.1371/journal.pone.0027621 PMC3215731

[17] Y. Dong , Essential protective role of tumor necrosis factor receptor 2 in neurodegeneration. Proc. Natl. Acad. Sci. U.S.A. 113, 12304–12309 (2016). 27791020 10.1073/pnas.1605195113 PMC5087045

[18] J. A. Hardy, G. A. Higgins, Alzheimer’s disease: The amyloid cascade hypothesis. Science 256, 184–185 (1992). 1566067 10.1126/science.1566067

[19] M. Ohno, Alzheimer’s therapy targeting the β-secretase enzyme BACE1: Benefits and potential limitations from the perspective of animal model studies. Brain Res. Bull. 126, 183–198 (2016). 27093940 10.1016/j.brainresbull.2016.04.007

[20] D. L. Herber , Microglial activation is required for Aβ clearance after intracranial injection of lipopolysaccharide in APP transgenic mice. J. Neuroimmune Pharmacol. 2, 222–231 (2007). 18040847 10.1007/s11481-007-9069-z

[21] F. Ginhoux, S. Lim, G. Hoeffel, D. Low, T. Huber, Origin and differentiation of microglia. Front. Cell. Neurosci. 7, 45 (2013). 23616747 10.3389/fncel.2013.00045 PMC3627983

[22] R. J. Andrew , Lack of BACE1 S-palmitoylation reduces amyloid burden and mitigates memory deficits in transgenic mouse models of Alzheimer’s disease. Proc. Natl. Acad. Sci. U.S.A. 114, E9665–E9674 (2017). 29078331 10.1073/pnas.1708568114 PMC5692556

[23] C. Pomilio , Microglial autophagy is impaired by prolonged exposure to β-amyloid peptides: Evidence from experimental models and Alzheimer’s disease patients. Geroscience 42, 613–632 (2020). 31975051 10.1007/s11357-020-00161-9 PMC7206478

[24] D. J. Selkoe, Soluble oligomers of the amyloid β-protein impair synaptic plasticity and behavior. Behav. Brain Res. 192, 106–113 (2008). 18359102 10.1016/j.bbr.2008.02.016 PMC2601528

[25] S. Sakaguchi, T. Yamaguchi, T. Nomura, M. Ono, Regulatory T cells and immune tolerance. Cell 133, 775–787 (2008). 18510923 10.1016/j.cell.2008.05.009

[26] Z. Ahmed , Actin-binding proteins coronin-1a and IBA-1 are effective microglial markers for immunohistochemistry. J. Histochem. Cytochem. 55, 687–700 (2007). 17341475 10.1369/jhc.6A7156.2007

[27] C. Zhu , Expression site of P2RY12 in residential microglial cells in astrocytomas correlates with M1 and M2 marker expression and tumor grade. Acta Neuropathol. Commun. 5, 4 (2017). 28073370 10.1186/s40478-016-0405-5 PMC5223388

[28] O. Butovsky , Identification of a unique TGF-β-dependent molecular and functional signature in microglia. Nat. Neurosci. 17, 131–143 (2014). 24316888 10.1038/nn.3599 PMC4066672

[29] A. Daria , Young microglia restore amyloid plaque clearance of aged microglia. EMBO J. 36, 583–603 (2017). 28007893 10.15252/embj.201694591 PMC5331757

[30] X. Guo, Y. Liu, D. Morgan, L. R. Zhao, Reparative effects of stem cell factor and granulocyte colony-stimulating factor in aged APP/PS1 mice. Aging Dis. 11, 1423–1443 (2020). 33269098 10.14336/AD.2020.0201 PMC7673847

[31] L. Marchetti, M. Klein, K. Schlett, K. Pfizenmaier, U. L. M. Eisel, Tumor necrosis factor (TNF)-mediated neuroprotection against glutamate-induced excitotoxicity is enhanced by *N*-methyl-d-aspartate receptor activation. Essential role of a TNF receptor 2-mediated phosphatidylinositol 3-kinase-dependent NF-κB pathway. J. Biol. Chem. 279, 32869–32881 (2004). 15155767 10.1074/jbc.M311766200

[32] M. A. Maia, E. Sousa, BACE-1 and γ-secretase as therapeutic targets for Alzheimer’s disease. Pharmaceuticals (Basel) 12, 41 (2019). 10.3390/ph12010041 PMC6469197 30893882

[33] X. L. He , Hydrogen sulfide down-regulates BACE1 and PS1 via activating PI3K/Akt pathway in the brain of APP/PS1 transgenic mouse. Pharmacol. Rep. 68, 975–982 (2016). 27372924 10.1016/j.pharep.2016.05.006

[34] Y. Y. Li , Icaritin improves memory and learning ability by decreasing BACE-1 expression and the Bax/Bcl-2 ratio in senescence-accelerated mouse prone 8 (SAMP8) mice. Evid. Based Complement. Alternat. Med. 2020, 8963845 (2020). 32714426 10.1155/2020/8963845 PMC7345953

[35] N. Kotagale , Involvement of hippocampal agmatine in β_1-42_ amyloid induced memory impairment, neuroinflammation and BDNF signaling disruption in mice. Neurotoxicology 80, 1–11 (2020). 32522471 10.1016/j.neuro.2020.06.002

[36] A. M. Wessels , Efficacy and safety of lanabecestat for treatment of early and mild Alzheimer disease: The AMARANTH and DAYBREAK-ALZ randomized clinical trials. JAMA Neurol. 77, 199–209 (2020). 31764959 10.1001/jamaneurol.2019.3988 PMC6902191

[37] M. F. Egan , Randomized trial of verubecestat for prodromal Alzheimer’s disease. N. Engl. J. Med. 380, 1408–1420 (2019). 30970186 10.1056/NEJMoa1812840 PMC6776078

[38] R. Qiu , Safety, tolerability, pharmacokinetics, and pharmacodynamic effects of PF-06751979, a potent and selective oral BACE1 inhibitor: Results from phase I studies in healthy adults and healthy older subjects. J. Alzheimers Dis. 71, 581–595 (2019). 31424395 10.3233/JAD-190228 PMC6839502

[39] D. Henley , Preliminary results of a trial of atabecestat in preclinical Alzheimer’s disease. N. Engl. J. Med. 380, 1483–1485 (2019). 30970197 10.1056/NEJMc1813435

[40] M. Chopra , Exogenous TNFR2 activation protects from acute GvHD via host T reg cell expansion. J. Exp. Med. 213, 1881–1900 (2016). 27526711 10.1084/jem.20151563 PMC4995078

[41] J. Koenigsknecht-Talboo, G. E. Landreth, Microglial phagocytosis induced by fibrillar beta-amyloid and IgGs are differentially regulated by proinflammatory cytokines. J. Neurosci. 25, 8240–8249 (2005). 16148231 10.1523/JNEUROSCI.1808-05.2005 PMC6725530

[42] C. Bellenguez ; EADB; GR@ACE; DEGESCO; EADI; GERAD; Demgene; FinnGen; ADGC; CHARGE, New insights into the genetic etiology of Alzheimer’s disease and related dementias. Nat. Genet. 54, 412–436 (2022). 35379992 10.1038/s41588-022-01024-z PMC9005347

[43] H. Gao , Opposing functions of microglial and macrophagic TNFR2 in the pathogenesis of experimental autoimmune encephalomyelitis. Cell Rep. 18, 198–212 (2017). 28052249 10.1016/j.celrep.2016.11.083 PMC5218601

[44] J. I. Rodriguez-Barbosa , The role of TNFR2 and DR3 in the in vivo expansion of Tregs in T cell depleting transplantation. Int. J. Mol. Sci. 21, 3347 (2020). 10.3390/ijms21093347 PMC7247540 32397343

[45] C. Dansokho , Regulatory T cells delay disease progression in Alzheimer-like pathology. Brain 139, 1237–1251 (2016). 26912648 10.1093/brain/awv408

[46] L. Zhong , Soluble TREM2 ameliorates pathological phenotypes by modulating microglial functions in an Alzheimer’s disease model. Nat. Commun. 10, 1–16 (2019). 30911003 10.1038/s41467-019-09118-9 PMC6433910

[47] T. F. Galatro , Transcriptomic analysis of purified human cortical microglia reveals age-associated changes. Nat. Neurosci. 20, 1162–1171 (2017). 28671693 10.1038/nn.4597

[48] H. Keren-Shaul , Unique microglia type associated with restricting development of Alzheimer’s disease. Cell 169, 1276–1290.e17 (2017). 28602351 10.1016/j.cell.2017.05.018

[49] A. Mildner, H. Huang, J. Radke, W. Stenzel, J. Priller, P2Y_12_ receptor is expressed on human microglia under physiological conditions throughout development and is sensitive to neuroinflammatory diseases. Glia 65, 375–387 (2017). 27862351 10.1002/glia.23097

[50] D. G. Walker , Patterns of expression of purinergic receptor P2RY12, a putative marker for non-activated microglia, in aged and Alzheimer’s disease brains. Int. J. Mol. Sci. 21, 678 (2020). 10.3390/ijms21020678 PMC7014248 31968618

[51] A. L. Hemonnot, J. Hua, L. Ulmann, H. Hirbec, Microglia in Alzheimer disease: Well-known targets and new opportunities. Front. Aging Neurosci. 11, 233 (2019). 31543810 10.3389/fnagi.2019.00233 PMC6730262

[52] E. E. Tuppo, H. R. Arias, The role of inflammation in Alzheimer’s disease. Int. J. Biochem. Cell Biol. 37, 289–305 (2005). 15474976 10.1016/j.biocel.2004.07.009

[53] H. Akiyama , Inflammation and Alzheimer’s disease. Neurobiol. Aging 21, 383–421 (2000). 10858586 10.1016/s0197-4580(00)00124-x PMC3887148

[54] K. J. Yin , Matrix metalloproteinases expressed by astrocytes mediate extracellular amyloid-beta peptide catabolism. J. Neurosci. 26, 10939–10948 (2006). 17065436 10.1523/JNEUROSCI.2085-06.2006 PMC6674654

[55] S. Rossner, C. Lange-Dohna, U. Zeitschel, J. R. Perez-Polo, Alzheimer’s disease β-secretase BACE1 is not a neuron-specific enzyme. J. Neurochem. 92, 226–234 (2005). 15663471 10.1111/j.1471-4159.2004.02857.x

[56] D. W. Dekens , Lipocalin 2 contributes to brain iron dysregulation but does not affect cognition, plaque load, and glial activation in the J20 Alzheimer mouse model. J. Neuroinflammation 15, 330 (2018). 30501637 10.1186/s12974-018-1372-5 PMC6267886

[57] W. Pan, A. J. Kastin, TNFα transport across the blood-brain barrier is abolished in receptor knockout mice. Exp. Neurol. 174, 193–200 (2002). 11922661 10.1006/exnr.2002.7871

[58] W. Pan, W. A. Banks, A. J. Kastin, Blood-brain barrier permeability to ebiratide and TNF in acute spinal cord injury. Exp. Neurol. 146, 367–373 (1997). 9270046 10.1006/exnr.1997.6533

[59] J. G. Vargas , A TNFR2-specific TNF fusion protein with improved in vivo activity. Front. Immunol. 13, 888274 (2022). 35769484 10.3389/fimmu.2022.888274 PMC9234581

[60] A. Fick, A. Wyzgol, H. Wajant, Production, purification, and characterization of scFv TNF ligand fusion proteins. Methods Mol Biol. 907, 597–609 (2012). 22907375 10.1007/978-1-61779-974-7_33

[61] L. Mucke , High-level neuronal expression of Aβ_1-42_ in wild-type human amyloid protein precursor transgenic mice: Synaptotoxicity without plaque formation. J. Neurosci. 20, 4050–4058 (2000). 10818140 10.1523/JNEUROSCI.20-11-04050.2000 PMC6772621

[62] A. A. Walf, C. A. Frye, The use of the elevated plus maze as an assay of anxiety-related behavior in rodents. Nat. Protoc. 2, 322–328 (2007). 17406592 10.1038/nprot.2007.44 PMC3623971

[63] A. Wolf, B. Bauer, E. L. Abner, T. Ashkenazy-Frolinger, A. M. S. Hartz, A comprehensive behavioral test battery to assess learning and memory in 129S6/Tg2576 mice. PLoS One 11, e0147733 (2016). 26808326 10.1371/journal.pone.0147733 PMC4726499

[64] D. Van Dam, G. Lenders, P. P. De Deyn, Effect of Morris water maze diameter on visual-spatial learning in different mouse strains. Neurobiol. Learn. Mem. 85, 164–172 (2006). 16290194 10.1016/j.nlm.2005.09.006

[65] I. B. Hovens, C. Nyakas, R. G. Schoemaker, A novel method for evaluating microglial activation using ionized calcium-binding adaptor protein-1 staining: Cell body to cell size ratio. Neuroimmunol. Neuroinflamm. 1, 82–88 (2014).

[66] E. De Jong, D. S. Williams, L. K. E. A. Abdelmohsen, J. C. M. Van Hest, I. S. Zuhorn, A filter-free blood-brain barrier model to quantitatively study transendothelial delivery of nanoparticles by fluorescence spectroscopy. J. Control. Release 289, 14–22 (2018). 30243824 10.1016/j.jconrel.2018.09.015

