# Supplementary material for: A TNF receptor 2 agonist ameliorates neuropathology and improves cognition in an Alzheimer’s disease mouse model
Source: Proc Natl Acad Sci U S A. 2022 Aug 29;119(37):e2201137119. doi: 10.1073/pnas.2201137119 (PMC9482428; doi:10.1073/pnas.2201137119)
Supplement: Supplementary File [file pnas.2201137119.sapp.pdf]

Supplementary Information for

A TNF Receptor 2 agonist ameliorates neuropathology and improves cognition in an Alzheimer’s disease mouse model

Natalia Ortí-Casañ[1], Inge S. Zuhorn[2], Petrus J. W. Naudé[1,3], Peter P. De Deyn[3], Pauline E.M. van Schaik[4], Harald Wajant[5] and Ulrich L. M. Eisel[1*]

[1]Department of Molecular Neurobiology, Groningen Institute for Evolutionary Life Sciences, University of Groningen, Groningen 9747 AG, Netherlands
[2]Department of Biomedical Engineering, University of Groningen, University Medical Center Groningen, Groningen 9713 AV, Netherlands
[3]Department of Neurology and Alzheimer Center, University of Groningen, University Medical Center Groningen, Groningen 9713 AV, Netherlands
[4]Department of Biomedical Sciences of Cells & Systems, Section Molecular Neurobiology, University of Groningen, University Medical Center Groningen, Groningen 9713 AV, the Netherlands.
[5]Department of Internal Medicine II, University of Würzburg, Würzburg 97070, Germany
*Correspondence and material requests should be addressed to U.E (u.l.m.eisel@rug.nl).

This PDF file includes:

Figures S1 to S5
Table S1

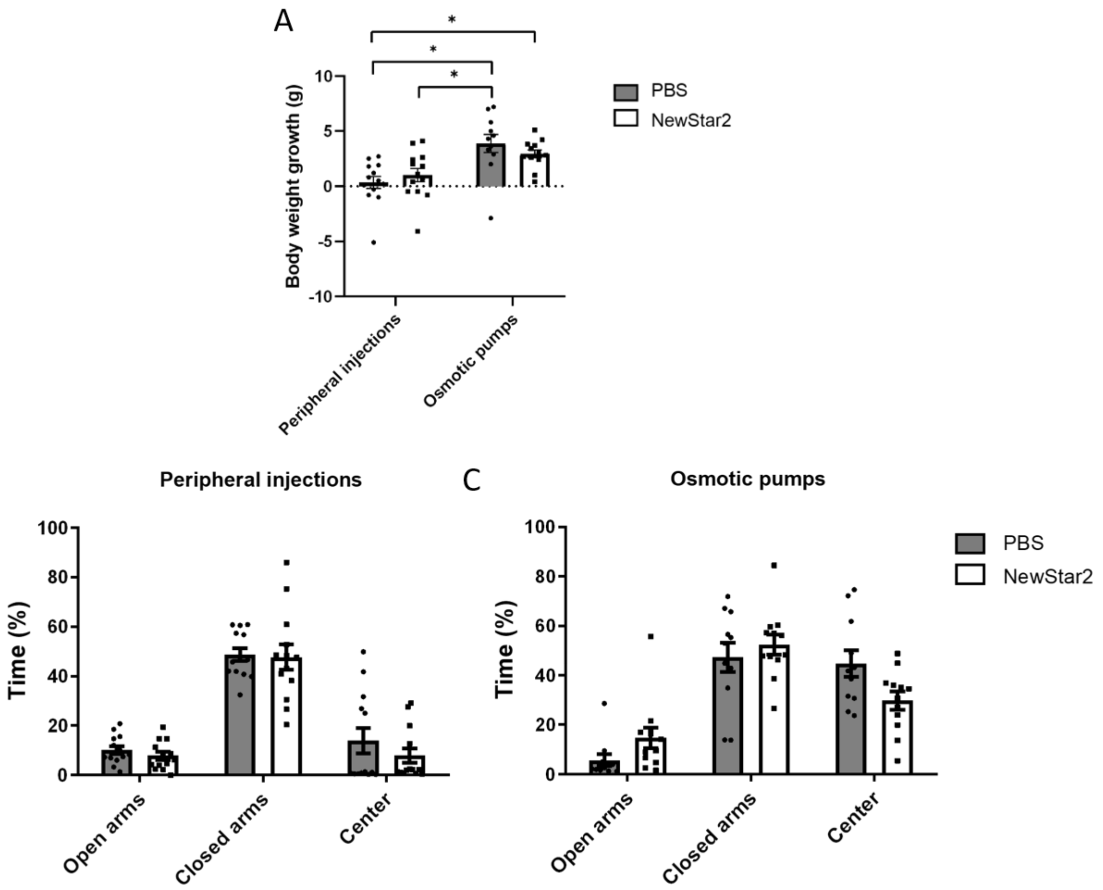

Fig. S1. NewStar2 does not affect body weight changes nor anxiety-like behavior. **A** NewStar2 was administered either systemically or centrally and body weight growth was measured (IP injections (PBS, n=13; NewStar2, n=13); Osmotic pumps (PBS, n=11; NewStar2, n=12)), two-way ANOVA, Bonferroni post hoc analysis ($F_{1, 45}$=20.21; DF=1). **B, C** Measurement of anxiety-like behavior in the EPM test (IP injections (PBS, n=13; NewStar2, n=13); two-way ANOVA, Bonferroni post hoc analysis ($F_{2,72}$=0.26; DF=2); Osmotic pumps (PBS, n=11; NewStar2, n=12), two-way ANOVA, Bonferroni post hoc analysis ($F_{2,63}$=4.33; DF=2)). Data are presented as mean ± SEM. *p<0.05

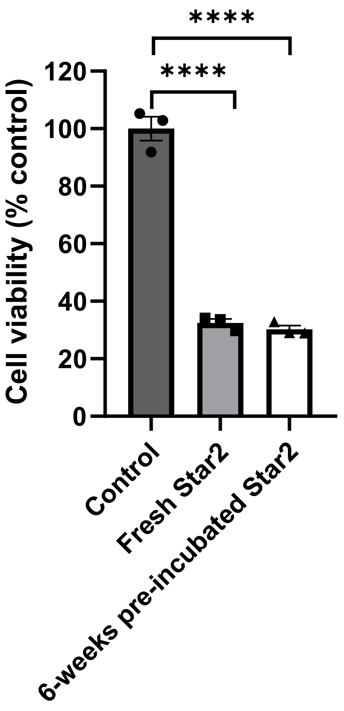

Fig. S2. Stability of NewStar2 is intact after 6-weeks incubation at 37°C. Fresh or 6-weeks pre-incubated NewStar2 was added to kym-1 cells for 24h and cell viability was measured using the crystal violet assay. Data are presented as mean ± SEM. Each condition was analyzed in triplicates, one-way ANOVA ($F_{2,6}$=227.22; DF=2). ****$p<0.0001$.

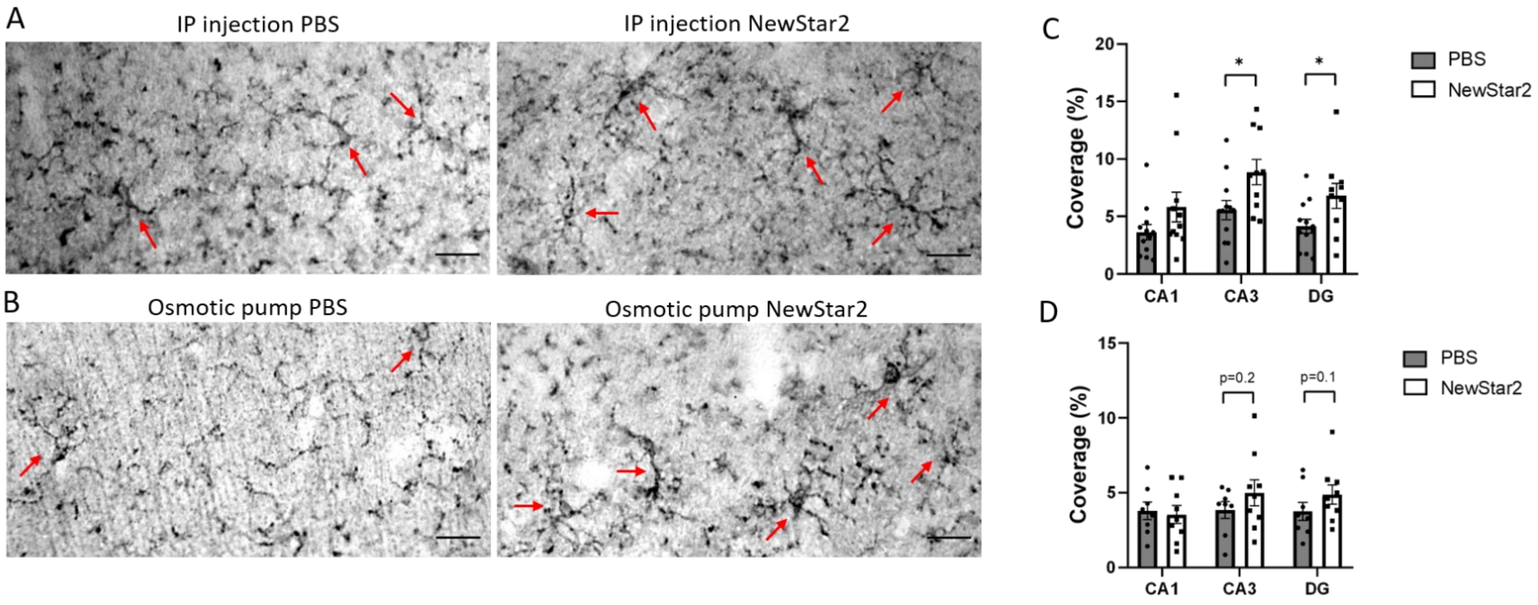

Fig. S3. Peripheral administration of NewStar2 leads to increased expression levels of P2RY12. **A** PBS vs. NewStar2 administration via IP injections or **B** osmotic pumps. Representative images of dentate gyrus are shown. Scale bar, 50 µm. **C** Quantification of resting microglia (P2RY12) coverage from CA1, CA3 and DG in **A** (IP injections (PBS, n=12; NewStar2, n=11), two-way ANOVA, Bonferroni post hoc analysis ($F_{1, 63}$=12.49; DF=1)). **D** Quantification of P2RY12 coverage from CA1, CA3 and DG in **B** (Osmotic pumps (PBS, n=8; NewStar2, n=9), two-way ANOVA, Bonferroni post hoc analysis ($F_{1, 45}$=1.54; DF=1)). Data are presented as mean ± SEM. *p<0.05.

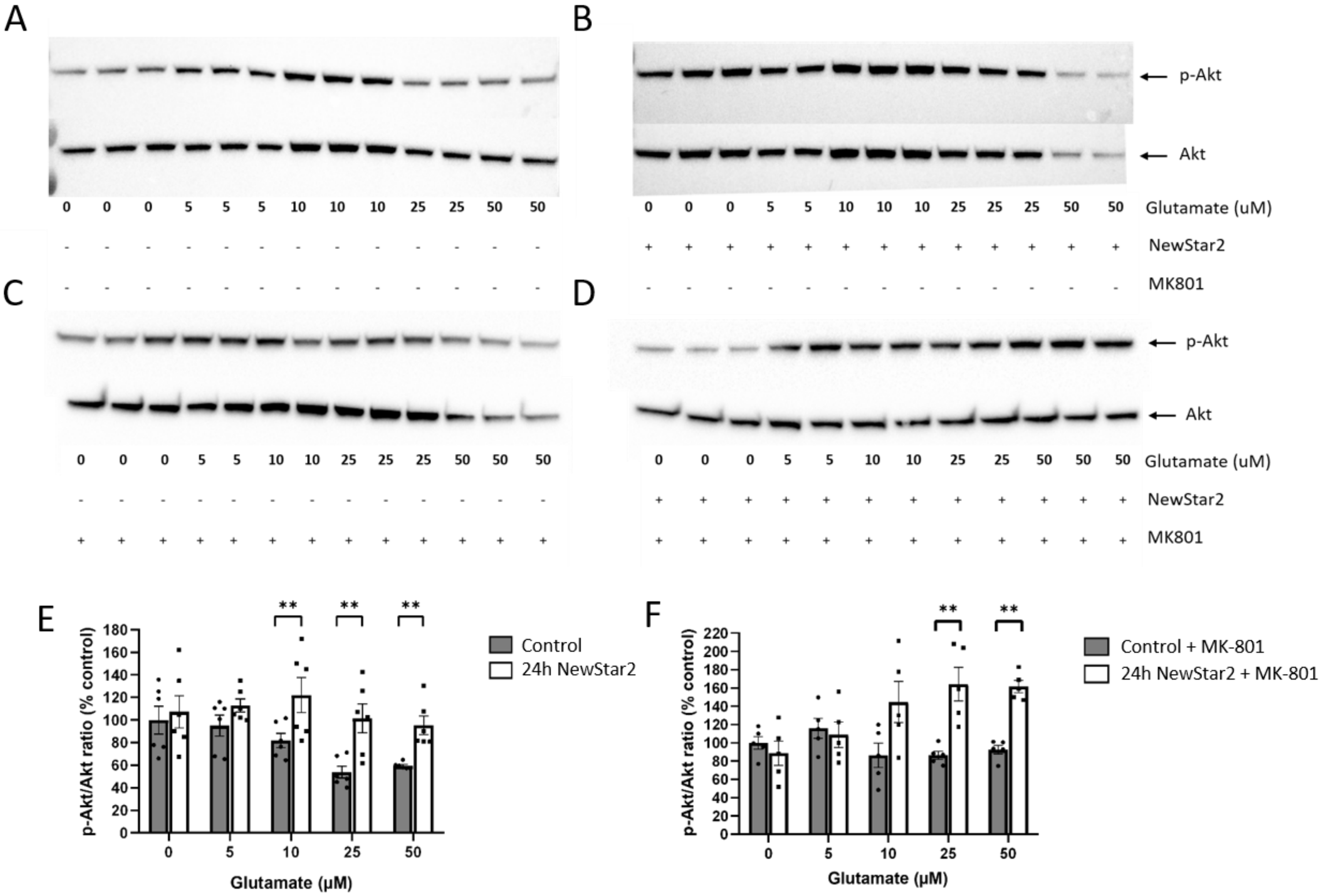

Fig. S4. Neuroprotective effect of NewStar2 in primary cortical neurons. Primary cortical neurons were pre-treated with NewStar2 for 24h and incubated with increasing toxic doses of glutamate with or without MK-801. PKB/Akt phosphorylation was measured in neuronal lysates using Western Blot. **A, B** Protein levels of p-Akt and Akt were measured by Western Blot in absence of MK801. **E** Quantification of p-Akt/Akt ratio (% control) from **A, B;** two-way ANOVA, Bonferroni post hoc analysis ($F_{1, 50}$=21.90; DF=1). **C, D** Protein levels of p-Akt and Akt were measured by Western Blot in presence of MK801. **F** Quantification of p-Akt/Akt ratio (% control) from **C, D**; two-way ANOVA, Bonferroni post hoc analysis ($F_{1, 40}$=17.96; DF=1). Data are presented as mean ± SEM. Each condition was analyzed in triplicates **(E)** or duplicates **(F)** from two different experiments. **p<0.01.

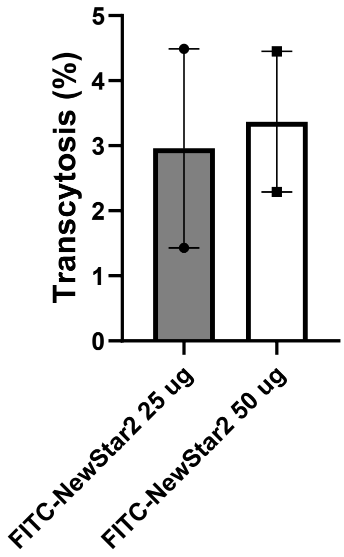

Fig. S5. NewStar2 crosses the blood-brain-barrier (BBB) in an *in vitro* BBB transcytosis model**.** 25 or 50 µg of FITC-NewStar2 was added apically to a hCMEC/D3 cells monolayer and the retrieved basal FITC-NewStar2 was used to calculate the percentage of transcytosis (%). Data are presented as mean ± SEM. Each condition was analyzed in duplicates from two different experiments.

Table S1. Primers used for genotyping of J20

Target	Forward	Reverse
J20 internal control	5'- CAA ATG TTG CTT GTC TGG TG-3'	5'- GTC AGT CGA GTG CAC AGT TT-3'
J20 transgene	5'- GGT GAG TTT GTA AGT GAT GCC-3'	5'- TCT TCT TCT TCC ACC TCA GC-3'
